# Molecularly Imprinted Sol-Gel for TNT Detection with Optical Micro-Ring Resonator Sensor Chips

**DOI:** 10.3390/s19183909

**Published:** 2019-09-10

**Authors:** Ludmila Eisner, Isabel Wilhelm, Günter Flachenecker, Jürgen Hürttlen, Wolfgang Schade

**Affiliations:** 1 Fraunhofer Heinrich-Hertz-Institute, Am Stollen 19H, 38640 Goslar, Germany; 2 Fraunhofer Institute for Chemical Technology, Joseph-von- Fraunhofer Strasse 7, 76327 Pfinztal, Germany; 3 Clausthal University of Technology, Am Stollen 19B, 38640 Goslar, Germany

**Keywords:** micro-ring resonator, molecularly imprinted sol-gel, evanescent field sensor, explosives

## Abstract

A sensor for trinitrotoluene (TNT) detection was developed by using a combination of optical micro-ring technology and a receptor coating based on molecularly imprinted sol-gel layers. Two techniques for deposition of receptor layers were compared: Airbrush technology and electrospray ionization. A concentration of less than 5 ppb for TNT in the gas-phase, using electrospray deposition of the receptor layer, was detected. The cross-sensitivities to organic substances and further nitro-based explosives were compared. As a result, the sensitivity to TNT is about one order of magnitude higher in comparison to the explosives 2,4-dinitrotoluene (DNT) or 1,3-dinitrobenzene (DNB) and about four orders of magnitude higher than the organic substances phenol, ethanol, and acetone. The signal response of the sensor is fast, and the compact sensor design enables the deposition of different receptor layers on multiple optical micro-rings on one chip, which allows a more precise analysis and reduction of side effects and false alarms.

## 1. Introduction

Early warning systems for risk prevention of terrorist attacks based on explosive detection at neuralgic public locations and infrastructures, such as airports, logistic centers, or governmental departments, are very important components of security management. For this, most existing technical approaches require a pre-selection of suspicious objects for detailed analysis. Fast detection of explosives in the gas-phase, stemming from contaminations at clothes or luggage, would be a significant enhancement for the recognition of explosives or other harmful chemicals used by terrorists. Therefore, miniaturized, portable, and reliable trace explosive detectors are needed. However, one of the current challenges for the detection of trace gas concentrations of explosives is the required sensitivity in the ppb range due to the low vapor pressures of most explosive substances. Additionally, the concurrent presence of interfering substances at comparably high concentrations complicates the analysis of the measurement. A variety of methods have been reported for detecting trinitrotoluene (TNT) in the gas-phase based on ion mobility spectrometry [1,2], surface acoustic wave devices [3], photoluminescence [4], electrochemistry [5,6], microcantilevers [7], and fluorescent polymers [8].

Most of the developed techniques for the detection of explosive devices are limited due to high cost, rather large size, and weight or limited reliability [9]. Gas-chromatograph mass spectroscopy (GC-MS) and ion-mobility spectroscopy (IMS) have a high analytical ability for the detection and quantification of explosives [10]. A drawback of gas-chromatography is the requirement of technical skills, a more extensive sample preparation, which makes a real-time analysis difficult. Additionally, the intrinsic system requirements for these analytical approaches complicate a miniaturization of the system. Better suited for mobile usage is IMS, but in comparison to GC-MS, IMS is more sensitive to side effects. Further, in the review article of Reference [10], most of the IMS systems are used in combination with a technical procedure for ablation of explosive molecules from contaminated surfaces and a subsequent analysis of the atmosphere with IMS in short distances.

Optical spectroscopic methods, such as absorption spectroscopy, laser-induced breakdown spectroscopy, and Raman spectroscopy, have shown analytical capabilities for the detection of explosives [11]. However, these spectroscopic methods are limited in sensitivity and have a low potential for miniaturization, otherwise the detection limit goes down or doesn’t offer capabilities for multispecies analysis. But the sensitivity can be increased by special sample preparation. For example, Zhu et al. [12] used hierarchically imprinted porous films for selective detection of explosives. In the presence of TNT or 2,4-dinitrotoluene (DNT) atmosphere, the fluorescence, stemming from the coating, changed and enabled the detection of gas-phase TNT and DNT at ppb concentrations of saturated vapor pressure. Surface-enhanced Raman scattering (SERS) of two picomolar TNTs in an aqueous solution was reported by Dasary et al. [13] by using cysteine-modified gold nanoparticles.

For miniaturization of optical concepts in photonics, integrated waveguide optics based on evanescent field interaction between the light guided within a waveguide and the surrounding medium are well-known for their high sensitivity, their miniaturized design, high mechanical stability, and cost efficiency, due to wafer-scale fabrication [14,15]. These sensors by themselves provide no detection selectivity but, instead, rely on specific adsorption properties by adding coatings or molecular surface layers on top of the waveguides. Further, the evanescent field interaction can be enhanced by resonant structures. Bharadwaj et al. [16] used a U-bend fiber optical sensor probe for evanescent field-based excitation of localized surface plasmon resonances (SPR) in combination with different receptors for detection of TNT and DNT in the gas phase. As an emerging sensing technology, optical micro-ring resonators have unique advantages in developing low-cost and portable vapor sensors [17]. A selective and sensitive detection of nitro-based explosives in the gas phase has successfully been demonstrated by using a photonic micro-ring resonator sensor coated with a receptor layer based on triphenylene-ketal with a supramolecular cage structure optimized for adsorption of TNT [18].

Receptor layers for miniaturized optical sensors are the key elements for sensitive and selective detection of explosives. Molecularly imprinted polymers (MIPs) principally have this advantage, offering specific molecular recognition sites, and can be used as selective recognition elements in sensors [19,20]. Selective detection of TNT without pre-separation by means of MIPs was already demonstrated using a mass sensitive readout [21] and other techniques [22,23], including electrochemical [24] and optical readouts via fiber or interferometer structures [25,26,27]. Nevertheless, with most of the described techniques, TNT concentrations in the lower ppb range are still hardly measurable. The requirements for the development of specific receptors for sensors in real applications are high, and the receptor coatings have to be reproducible and stable for a longer time. Further, the adaption of receptors for new explosives should be feasible in a short time, and standard procedures for the production of receptors and simple variation of chemical affine structures with less effort for development are preferred. Therefore, the approach followed in this work is the combination of selective sol-gel MIPs with a very sensitive optical evanescent field sensor for demonstration. We used optical micro-ring resonators for the measurement of wavelength shifts due to evanescent interactions. 2,4,6-trinitrotoluene was chosen as the target substance because it is one of the most commonly used high explosives, which not only represents a security threat, but is also of environmental concern due to soil and water contamination.

## 2. Materials and Methods

### 2.1. Sol-Gel Materials

**Figure 1:**
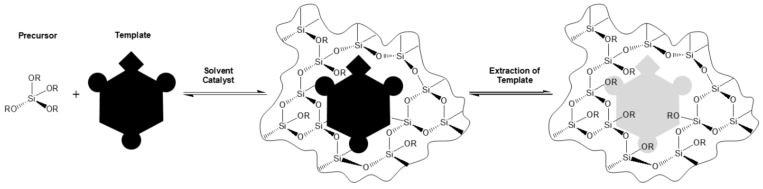
Scheme of sol-gel imprinting process.

The sol-gel process is a low temperature-method for producing ceramics and organic-inorganic hybrids. In general, it is a process forming a colloidal liquid (sol) into a network (gel). The precursors used for preparation of the colloid are usually metalorganic compounds (like metal alkoxides) or inorganic metal salts. The reaction is accomplished by hydrolysis and polycondensation to form a sol-gel-network (Figure 1).

During this process, the viscosity of the solution increases. The by-products, such as alcohols or water, can be easily removed by a drying process. Wet chemical coating processes, such as dip-, spray- and spin-coating, can be used to produce porous films from sols.

The growing interest in sol-gel materials is due to their excellent properties in the use of molecular imprinting. To design artificial antibodies for molecular recognition, a template is added to the sol-gel matrix. The imprinted sol-gel material with desired features is chemically, photochemically, and thermally more stable compared to an organic molecularly imprinted polymer [28,29,30,31].

### 2.2. Principle of Sensing

The sensors were based on a straight ridge waveguide, connected with a coherent light source at the input and a photodetector at the output. Additionally, a resonant cavity, consisting of a ridge waveguide in form of a ring-type structure, was placed very close to the straight waveguide. The gap between the straight and the ring waveguides was sufficiently small for realizing an overlap of the evanescent light fields leaking out of the waveguides and thus enabled an exchange of energy between them. The cavity mode in the ring waveguide was excited by evanescent coupling between the ring and the straight waveguide. If the guided light in the ring had a round trip phase shift of integer times 2*π*, the cavity was in resonance and the light field built up. By coupling back to the straight waveguide, the light was phase shifted. This phase shift depended on the coupling conditions between the straight and the ring waveguides and the intensity of the light at the output decreases, due to excessive destructive interference, if the wavelength of light fulfills the following resonance condition in the ring waveguides.

(1) $$m\;\lambda =2\pi r\;n_{eff},\;\;m=1,2,3,\ldots$$

where *m* is an integer, λ is the wavelength of light, *r* is the radius of the ring, and *n_eff_* is the effective refractive index of the ring waveguide. The interaction of the evanescent field overlapping with the surrounding media is influencing the effective refractive index. According to Equation (1), this leads to a median dependent shift of the resonant wavelength:

(2) $$m\;\left(\lambda +\Delta \lambda \right)=2\pi r\left(n_{eff}+\Delta n_{eff}\right)$$

where ∆*n_eff_* is the change of the effective refractive index and ∆λ is the change of the resonant wavelength. Dividing Equations (2) by (1) results in:

(3) $$\frac{n_{eff}+\Delta n_{eff}}{n_{eff}}=\frac{\lambda +\Delta \lambda }{\lambda }\;\mathrm{or}\;\frac{\Delta n_{eff}}{n_{eff}}=\frac{\Delta \lambda }{\lambda }$$

Consequently, the change of the resonant wavelength was dependent on the change of the refractive index of the environment. These measurements were not chemically specific, because any change of the composition of the substances in the gas phase or solution can contribute to a change of the effective refractive index and consequently to the resonant wavelength shift.

To solve this problem, the micro-ring surface was covered with a receptor layer, which bonded the analyte molecules specifically on the sensor surface, resulting in a significant shift of resonance wavelength. This enabled a more selective detection, but side effects by other substances still had to be considered.

### 2.3. Sensor Design

The optical structures of the sensor chip were fabricated using standard plasma-enhanced chemical vapor deposition (PECVD) techniques on a silicon-on-insulator wafer with a 5 µm silicon oxide layer and a 250 nm silicon nitride layer.

The sensor chip contained race-track-shaped micro-ring resonators designed with a 1.25 µm waveguide, a 40 µm coupling length, and a 200 µm radius, achieving a free spectral range (FSR) of 0.38 nm and a Q-factor of about 37,800.

**Figure 2:**
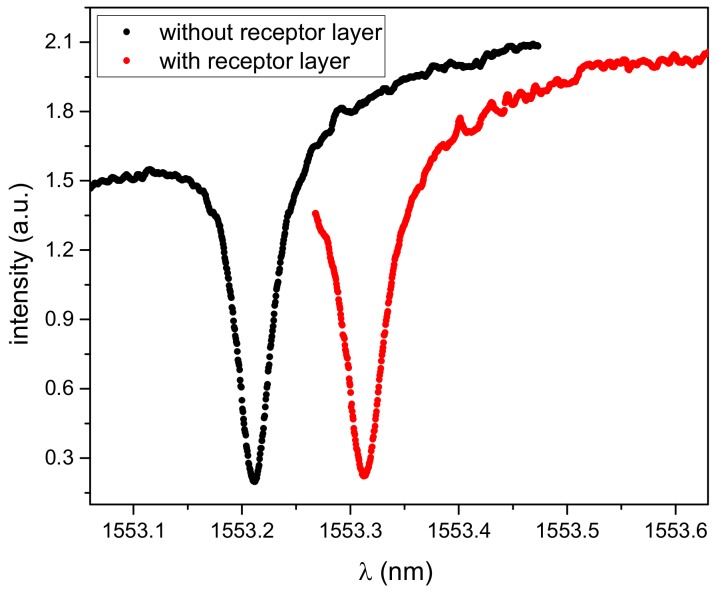
Typical transmission spectrum of a micro-ring resonator. Black: transmission spectrum of the micro-ring resonator without receptor layer. Red: transmission spectrum of the micro-ring resonator with receptor layer.

The design of the micro-ring resonator array chip consisted of a waveguide connected to a fiber-coupled coherent light source. This waveguide was split into 4 parallel branches with micro-rings. The transmission of each branch could be measured independently. Figure 2 shows a typical transmission spectrum of a micro-ring resonator (black line).

**Figure 3:**
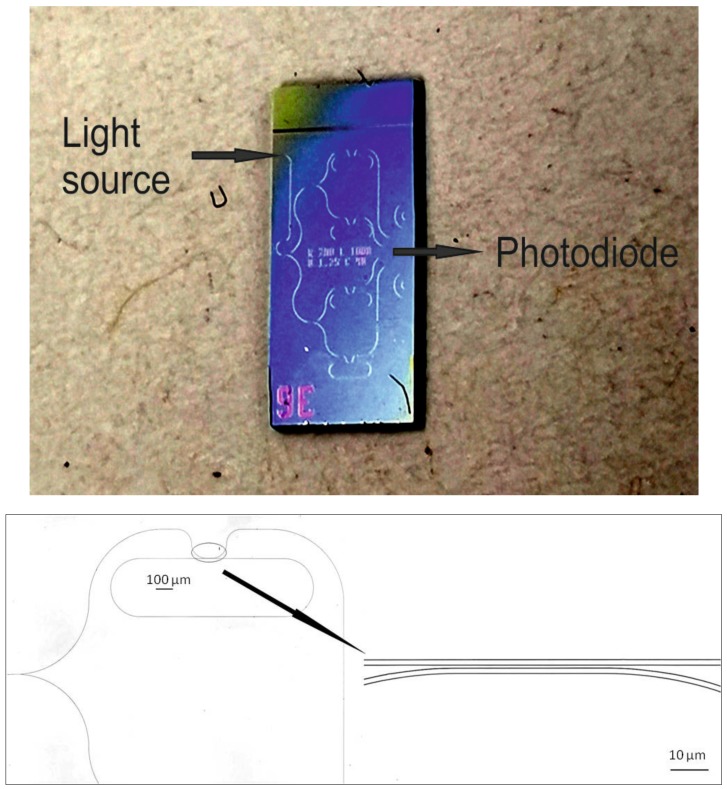
Photo of the complete sensor chip (top) and laser scanning microscope image of one micro-ring (bottom).

A photo of the whole chip and the waveguide design around a micro-ring resonator, recorded with a laser scanning microscope (Keyence VK-X200), is shown in Figure 3.

The use of several ring resonators coated with different kinds of receptors allowed multi-species detection to be simplified. One of the micro-rings was utilized as a reference ring for compensation of temperature-induced shifts of resonance wavelength and was not coated with any receptor.

After covering with the receptor layer, the resonant peak was broadened and shifted towards longer wavelengths due to a refractive index change (Figure 2, red line). The Q-Factor of the covered micro-ring resonator decreased slightly to 32,700.

### 2.4. Receptors

#### 2.4.1. Reagents

3,5-Dinitrobenzyl alcohol, 3-isocyanatopropyltriethoxysilane, and tetrabutylammonium fluoride were obtained from Sigma-Aldrich (Schnelldorf, Germany). 2-(2-Pyridylethyl)trimethoxysilane and bis(trimethoxysilylethyl)benzene were obtained from abcr (Karlsruhe, Germany). Tetrahydrofuran (puriss., p.a.) was obtained from Honeywell (Offenbach, Germany).

#### 2.4.2. Template Synthesis

**Figure 4:**
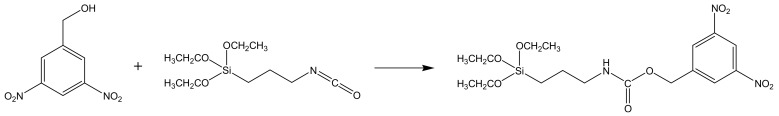
Scheme of template synthesis.

For the imprinting effect for TNT, the generation of amine groups as hydrogen bond donators for the nitro groups is essential. In contrast to the radical polymerization for acrylate-based MIPs, these amino-groups could not be synthesized without protection during the sol-gel formation. Therefore, a special carbamate was used so that the cleavage of the carbamate linkage yielded in binding sites with amine groups. A template (Figure 4) and sol-gel molecularly imprinted matrix were synthesized, following the method described by Walker et. al. [25], with some minor modifications. Briefly, 1.0 g (5 mmol) of 3,5-dinitrobenzylalcohol was dissolved in 0.3 mL tetrahydrofuran. A total of 1.25 mL (5 mmol) 3-isocyanatopropyltriethoxysilane was added drop wise. The mixture was stirred 36 h at reflux (60 °C) under nitrogen atmosphere. A yellow light-brown product was obtained and used for the synthesis of imprinted sol-gel without further purification. 

The product was confirmed by ^1^H and ^13^C NMR spectroscopy. 400MHz ^1^H NMR (DMSO-d6) δ (ppm): 8.76 (s, 1H, aromatic); 8.62 (s, 2H, aromatic); 7.53 (s, 1H, N*H*); 5.28 (s, 2H, Ph–C*H*_2_OC=O); 3.74 (q, 6H, Si–OC*H*_2_CH_3_); 2.99 (q, 2H, C*H*_2_NH); 1.30

(m, 2H, CH_2_C*H*_2_CH_2_; 1.14 (m, 9H, Si–OCH_2_C*H*_3_); 0.53 (t, 2H, Si–C*H*_2_); and ^13^C NMR (DMSO-d6) δ (ppm): 156.2 (O*C*=ONH); 148.1 (C3 phenyl); 142.2 (C1 phenyl); 127.4 (C2 phenyl); 117.7 (C4 phenyl); 63.2 (O*C*H_2_–Ph); 57.8 (Si–O*C*H_2_CH_3_); 43.1 (NH*C*H_2_); 25.1 (NHCH_2_*C*H_2_); 18.2 (Si–OCH_2_*C*H_3_); 7.2 (Si–*C*H_2_).

#### 2.4.3. Synthesis of Imprinted Sol-Gel

For synthesis of sol-gel MIP, 0.94 mL (2.7 × 10^−3^ mol) bis(trimethoxysilylethyl)benzene, 0.06 mL (3.0 × 10^−4^ mol) 2-(2-pyridylethyl)trimethoxysilane, and 0.02 g (4.5 × 10^−5^ mol) templates were added to 200 mL tetrahydrofuran. A mixture of 0.33 mL H_2_O and 0.3 mL 1 M tetrabutylammonium fluoride (in tetrahydrofuran) was slowly added to this solution. The whole solution was aged while stirring at room temperature for 5 days. Non-imprinted sol-gel was synthesized the same way, except the template was not added to the solution.

#### 2.4.4. Deposition of Receptor Coating

Two coating processes of the sensor chips were used: Airbrush coating and electrospray ionization [32,33]. For both coating processes, special masks were used for selective deposition of the sol-gel MIP receptor layer on one individual micro-ring on the chip. We coated the second micro-ring with non-imprinted sol-gel for comparison and the third micro-ring remained uncovered for monitoring of temperature effects. The fourth ring was not used. During the coating process, we monitored the intensity and width of the resonant dip of the transmission signal for controlling the optimum layer thickness in real time. This was necessary, because the evanescent field of the light, guided in the waveguides, decreased rapidly within a view hundred nanometers. If the coating layer was too thick, the interaction with the adsorbed molecular analytes was not possible anymore. Due to the deposition of the coating, a shift of the wavelength depending on the thickness of the layer occurred. We stopped the deposition process when the shift of resonant wavelength reached the maximum. The resulting layer thickness was estimated based on SEM images of 500–700 nm. 

The coated micro-ring resonators were rinsed with 1 M HCl solution, followed by chloroform, and dried in air to remove the template molecule.

**Figure 5:**
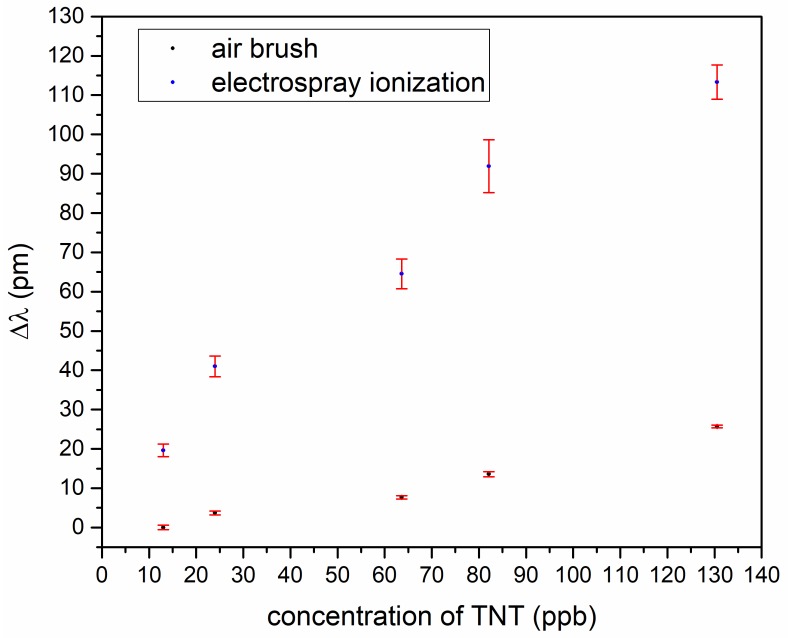
Shift of the resonant wavelength in dependence of trinitrotoluene (TNT) concentration. Blue: Chip was covered by air brush proceeding, black: Chip was covered by electrospray ionization.

To find out the best-suited coating process, we made a TNT measurement series for comparison of the airbrush and electrospray ionization. Figure 5 shows a wavelength shift in dependence of TNT concentration and, as can be seen, the electrospray coating was better. For the further experimental series, electrospray ionization was used.

## 3. Results

**Figure 6:**
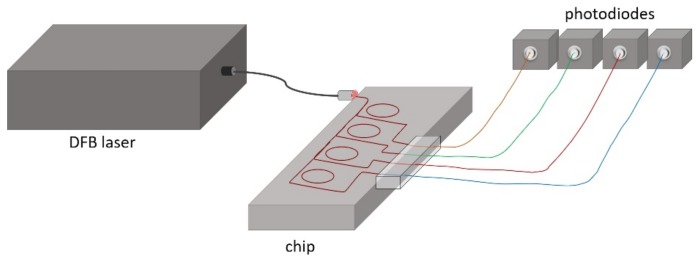
Schematic configuration of the experimental set up.

The experimental setup included a tunable distributed feedback (DFB) laser diode with central wavelength at 1.552 µm and a spectral line width of 1 MHz, which was connected to the micro-ring chip via a single-mode fiber. Spectral tuning of the wavelength was done by applying a voltage ramp to a piezo actuator. Since the emission wavelength depended on both the temperature of the diode and the applied voltage, an accurate control of both parameters was essential. For temperature stabilization, a closed loop control algorithm was developed. The voltage was correlated to the wavelength by a calibration data set for each temperature. Four InGaAs photodiodes (PDA 10SC-ES from Thorlabs Inc.) were used for the measurement of the transmitted intensity. Figure 6 shows the schematic view of the experimental setup.

For the measurement of explosives at different temperatures, the TNT, DNT, or 1,3-dinitrobenzene (DNB) samples were put together with the sensor chip into an open glass container and sealed with parafilm tapes. We put this container into a water bath for achieving a definite temperature within the glass container. The water bath temperature was adjusted by using a heating plate with temperature control from Heidolph instruments ( type: MR Hei-standard). 

The sol-gel MIP-coated micro-ring sensors were characterized for different analytes: The explosives trinitrotoluene (TNT), 2,4-dinitrotoluene (DNT), and 1,3-dinitrobenzene (DNB), and the aromatic and non-aromatic solvents phenol, acetone, and ethanol. We selected these solvents because they are very often present in public rooms and potentially interfere with the measurement of the explosives, due to a similar chemical structure or polarity.

One micro-ring was coated with a sol-gel MIP layer, which is tailored for strong adsorption of TNT molecules. For comparison, a second micro-ring was coated with a non-imprinted sol-gel polymer layer for monitoring the signal changes induced by adsorption of molecules. Temperature changes affected all micro-rings equally. Therefore, an uncoated micro-ring was used for simultaneous reference measurements in order to consider temperature-induced effects of signal changes. After exposing the sensor to TNT vapor or other analytes, the coated micro-ring resonators were rinsed with chloroform and dried in air to remove the adsorbed molecules. For testing the sensitivity of the sol-gel MIP-coated micro-ring sensor, we generated different concentrations of TNT, DNT, and DNB in air by stepwise heating the sample in a closed volume of a sample chamber, considering that, after a sufficient time at constant temperature, equilibrium vapor pressure of TNT in the atmosphere originates. The vapor pressure of explosives in dependence of the temperature is well known from literature [34,35,36,37]. 

Before starting the series of measurements of variable TNT, DNT, and DNB concentrations in air, the wavelength of the resonance peak for transmission losses was recorded without the analytes in the atmosphere at room temperature. For the subsequent measurements of the analytes, the test sample was placed in the vicinity of the micro-ring array chip inside a closed temperature-controlled glass container. We started at room temperature and increased the temperature from 5 °C, stepping up to 55 °C. 

**Figure 7:**
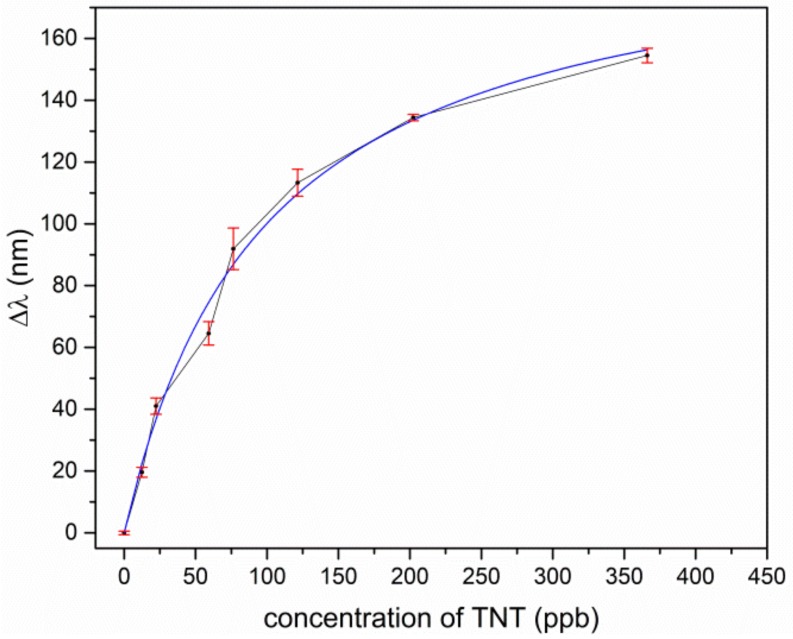
Shift of the resonant transmission minimum to a longer wavelength in dependence of TNT concentration.

**Figure 8:**
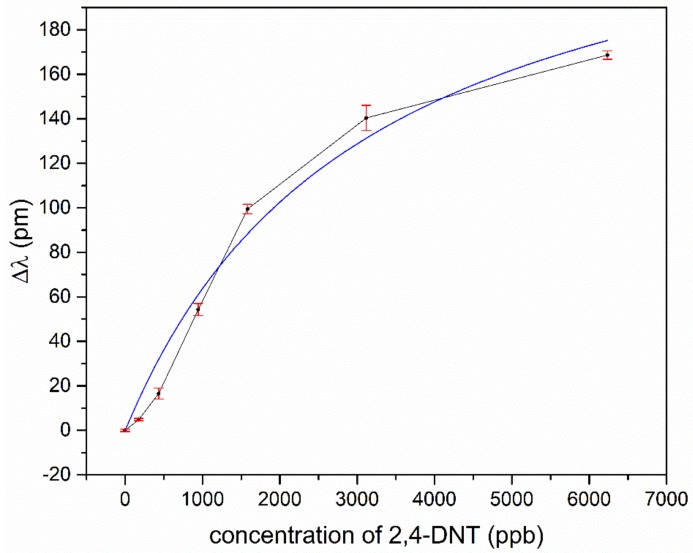
Shift of the resonant transmission minimum to a longer wavelength in dependence of DNT concentration.

For TNT and TNT derivatives, the imprinted polymer layer offerred many tailored docking positions, where the analyte was preferably deposited and therefore enriched in the coating layer. This resulted in an increased refractive index in the coating material, and, due to the interaction of the leaking evanescent field of the light, it was guided in the ring waveguide and the overall effective refractive index of the ring waveguide also changed. This shifted the wavelength of transmission minimum towards a longer wavelength. In Figure 7 and Figure 8, for TNT and DNT, the shifts of the resonant wavelengths of the sol-gel MIP-coated micro-rings, depending on the concentration of explosives, are displayed. 

For low concentrations, the response of the sensor is approximately linear for TNT and DNT. A saturation effect comes up for TNT at concentrations above 150 ppb and for DNT above 2000 ppb. Assuming the effect of the different temperatures in our experiments to play a minor role for adsorption kinetics, we fitted our data according to Langmuir’s empirical isothermal adsorption model. Within this approach, the adsorption can only occur at a finite (fixed) number of definite localized sites which are identical and equivalent, with no lateral interaction and steric hindrance between the adsorbed molecules [38]. As can be seen, Langmuir’s model fits almost perfectly to the experimental data of TNT adsorption, as in Figure 7, but the slightly s-shaped course of the experimental data for DNT in Figure 8 is not represented sufficiently. Typically, such an s-shaped course in isothermal adsorption curves arises by multilayer adsorption of molecules, due to capillary condensation in meso- and micropores. The vapor pressure for TNT is much lower in comparison to DNT, and the tailored docking sites are ideally isolated host-guest sites for single TNT molecules, which in sum fulfills the assumptions of the Langmuir model. This no longer holds for DNT, due to a higher vapor pressure and less strong binding affinities to the imprinted host-guest sites in the polymer. Moreover, the nano- and microporous structure of the coating promotes capillary condensation even outside the host-guest sides. 

**Figure 9:**
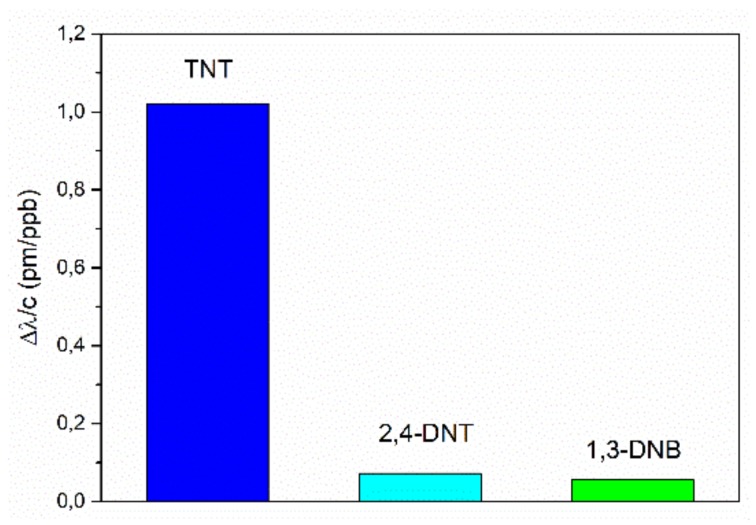
Shift of the resonant wavelength for 1 ppb concentration of explosive.

**Figure 10:**
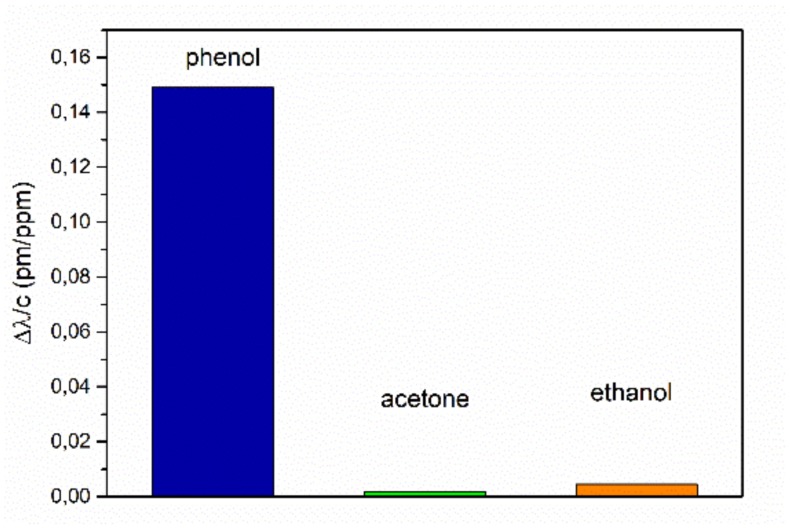
Shift of the resonant wavelength for 1 ppb concentration of organic solvents.

For comparison of the sensor’s sensitivities of the investigated explosives, we displayed the constant sensitivities for the linear sensor range at low concentrations, respectively, in Figure 9. The sensitivity for TNT (1.02 ppb/pm) is more than 14 times higher in comparison to the sensitivities for DNT (0.07 ppb/pm) and DNB (0.06 pm/ppb) detection. In addition, the response of the micro-ring with a non-imprinted polymer layer to TNT and DNT was investigated. The micro-ring resonator showed no sensitivity either to TNT or DNT. The higher response for TNT may have been caused by the formation of a Janovsky complex [39], where TNT is deprotonated at the methyl group by the amino groups in the cavity to form an anion. This leads to a higher change of the refractive index of the sol-gel material. The smaller wave length shift for DNT may indicate that the amine group is not basic enough to deprotonate the methyl group of DNT. For testing the cross-sensitivity, we measured the signal changes from pure air atmosphere to the saturated vapor pressure atmospheres of the organic substances, phenol, acetone, and ethanol, at room temperature. The sensitivities derived from these measurements are shown in Figure 10. The sensitivities for these organic substances are about three orders of magnitudes smaller than for the explosives, with 0.149 ppm/pm for phenol, 0.0019 ppm/pm for acetone, and 0.0045 ppm/pm for ethanol.

## 4. Discussion

Thin layers of sol-gel-imprinted polymers imprinted for TNT adsorption were deposited on top of highly sensitive photonic micro-ring chips. In comparison to micro-ring chips prepared with sol-gel non-imprinted polymers, a selective signal change was induced in the gas phase TNT atmosphere, and concentrations of about 5 ppb were detected. It was shown that TNT imprinted polymer layers are also suited for the detection of the derivatives DNT and DNB. The sensitivity of DNT and DNB is less than TNT, but due to their high partial pressures in comparison to TNT, these derivatives were also detected. The sensitivity for the other organic substances (phenol, acetone, and ethanol) at room temperature was demonstrated to be much lower. However, due to high partial pressures, the concentrations of such organic substances can be highly concentrated in air and may be responsible for unwanted cross side effects of a single sensor. Therefore, multiple sensors with different receptors had to be used for the identification of individual explosives. By fabrication and deposition of tailored layers of imprinted polymers for a set of sensors, the evaluation of all signals can be used for a more precise analysis of the atmosphere. In summary, we demonstrated a sol-gel process for the production of imprinted polymers in combination with an optical evanescent field detector to be well suited for sensitive detection of nitro-based explosives. Further, these receptor layers have a fast response and are refreshable. For future applications, a set of sensors with different imprinted polymers have to be prepared, and the collective signal response from the sensors should be evaluated by pattern recognition, trained with neuronal networks.
